# Potentials of the elevated circulating miR-185 level as a biomarker for early diagnosis of HBV-related liver fibrosis

**DOI:** 10.1038/srep34157

**Published:** 2016-09-28

**Authors:** Bin-bin Li, Dong-liang Li, Chao Chen, Bao-hai Liu, Chun-yan Xia, Han-jun Wu, Chao-qun Wu, Guo-qin Ji, Su Liu, Wu Ni, Ding-kang Yao, Zhi-yu Zeng, Da-gui Chen, Bao-dong Qin, Xuan Xin, Gang-li Yan, Dan Tang, Hui-min Liu, Jin He, Hongli Yan, Wei-Jian Zhu, Hong-yu Yu, Liang Zhu

**Affiliations:** 1 Department of Pathology, Changzheng Hospital, Second Military Medical University, Shanghai 200433, China; 2 Department of Gastroenterology, Changzheng Hospital, Second Military Medical University, Shanghai 200433, China; 3 Department of Hepatobiliary Medicine, Fuzhou General Hospital of Nanjing Military Command, Fuzhou 350025, Fujian Province, China; 4 Department of Gastroenterology, the First Affiliated Hospital of Chinese PLA General Hospital, Beijing 100037, China; 5 Department of Training, the Second Military Medical University, Shanghai 200433, China; 6 Department of Gastroenterology, Shanghai First People’s Hospital, Shanghai Jiaotong University School of Medicine, Shanghai 200003, China; 7 School of Life Sciences, Fudan University, Shanghai 200433, P. R. China; 8 State Key Laboratory of Genetic Engineering, Institute of Genetics, School of Life Sciences, Fudan University, Shanghai 200433, P. R. China; 9 Institutes of Biomedical Sciences, Fudan University, Shanghai, 200032, P. R. China; 10 Department of Gastroenterology, the Second Military Medical University, Shanghai 200433, China; 11 Department of Infectious Diseases, the Second Military Medical University, Shanghai 200433, China; 12 Department of Pharmacy, College of Pharmacy, Changzheng Hospital, Second Military Medical University, Shanghai 200433, China; 13 Department of Clinical Laboratory, Changzheng Hospital, Second Military Medical University, Shanghai 200433, China; 14 Department of Laboratory Medicine Changhai Hospital, The Second Military Medical University, 168 Changhai Road, Shanghai 200433, P. R. China

## Abstract

Early diagnosis of liver fibrosis is critical for early intervention and prognosis of various chronic liver diseases. Conventional repeated histological assessment is impractical due to the associated invasiveness. In the current study, we evaluated circulating miR-185 as a potential biomarker to predict initiation and progression of liver fibrosis. We found that miR-185 was significantly up-regulated in blood specimens from patients with HBV-liver fibrosis and rats with liver fibrosis, the miR-185 levels were correlated with liver fibrosis progression, but not with the different viral loads in HBV-infected patients. miR-185 was observed in collagen deposition regions during advanced liver fibrosis. We found that differences in miR-185 levels facilitated the discrimination between early-staged or advanced-staged liver fibrosis and the healthy controls with high specificity, sensitivity, and likelihood ratio using receiver-operator characteristic analysis. miR-185 targeted SREBF1, and increased expression of COL1A1 and a-SMA genes that are hallmarks of liver fibrosis. Our data supported that circulating miR-185 levels could be used as potential biomarkers for the early diagnosis of liver fibrosis.

Liver fibrosis is a major cause of morbidity and mortality worldwide^1^,^2^. It can progress to liver cirrhosis and cancer, or liver failure in pro-tumorigenic microenvironments. Studies have shown that liver fibrosis can be reversed to normal status, but liver cirrhosis cannot be reversed^3^,^4^. Prevention or reversal of liver fibrosis at an early stage can improve the prognosis of various chronic liver diseases^5^,^6^. Early diagnosis of liver fibrosis is a critical step for early intervention.

Currently, histological assessment is the gold standard approach for diagnosis of liver fibrosis. Studies have shown that repeated histological assessments for high-risk patients undergoing intervention with antiviral treatment are helpful in tracing the dynamic changes in fibrotic burden and predicting long-term prognosis more accurately^7^,^8^. However, this method is impractical, primarily owing to the inherent invasiveness of liver biopsies. This also renders the method unsuitable for evaluating the effect of therapy shortly after treatment^9^,^10^. Therefore, it is imperative to find an appropriate surrogate biomarker for identifying liver fibrosis at early stages of development. This would also allow monitoring of liver fibrosis progression by non-invasive follow-up methods, which is very important for initiating earlier interventions.

miRNAs(MicroRNAs) are short, non-coding RNAs consisting of approximately 22 nucleotides that negatively regulate the expression of protein-coding genes. It has been well documented that miRNAs circulate in a stable and detectable form with minimal inter-individual variability^11^,^12^. Studies have shown that miRNAs can be secreted into the extracellular milieu in response to inflammation or hepatocyte injury^13^, and circulating miRNAs can exhibit distinct expression patterns in human chronic hepatic fibrosis^14^. Individual miRNAs have been identified as biomarkers for inflammation in various forms of liver injury^15^. The circulating miRNAs, miR-29a and miR-92a, have been shown to be effective noninvasive biomarkers for human colorectal cancer. MiR-208a has been shown to be an effective noninvasive biomarker for acute myocardial infarction in humans^16^,^17^. Therefore, miRNAs might be a good candidate biomarker for early detection and staging of liver fibrosis.

In the present study, we characterized miR-185 as a potential candidate to predict initiation and progression of liver fibrosis using rat liver fibrosis models and specimens from patients. We found that miR-185 was significantly up-regulated in both human blood specimens and the fibrotic rat blood specimens, and the magnitude of increases in the miR-185 levels is correlated with the progression of liver fibrosis but not with different viral loads in HBV-infected patients. miR-185 was observed in collagen deposition regions during advanced liver fibrosis. The miR-185 levels can discriminate between early-staged and advanced-staged liver fibrosis in the healthy controls with high specificity, sensitivity, and likelihood ratio. Furthermore, miR-185 targeted SREBF1 and increased expression of COL1A1 and a-SMA genes, which are hallmarks of liver fibrosis. Our data supported the hypothesis that the level of circulating miR-185 was probably a potential candidate for early diagnosis of liver fibrosis, leading to early intervention and reversal of liver fibrosis.

## Results

### Circulating miR-185 is up-regulated in both the dimethylnitrosamine(DMN) and bile duct ligation(BDL) rat models

**Figure 1:**
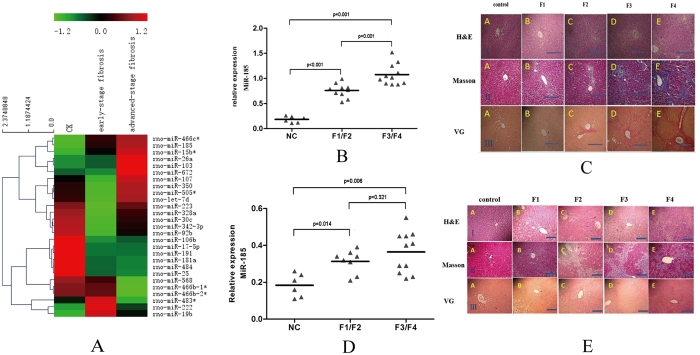
Blood miR-185 levels in rats with liver fibrosis induced by DMN and BDL. (**A**) Differentially-changed blood miRNA levels in DMN liver fibrosis rat model. The miRNA levels were determined using microRNA array and validated by qRT-PCR. (**B**) Blood miR-185 levels in normal rats (NC, n = 6), rats with DMN-induced early-stage liver fibrosis (n = 10), and rats with advanced-stage liver fibrosis (n = 11). The line indicates the median value in each group. Fold-changes were calculated using the 2^-ΔΔCt^ method. The Mann-Whitney test was used as the statistical significance test. (**C**) Histological confirmation of rat liver fibrosis induced by DMN. (**D**) Blood miR-185 levels in normal rats (NC, n = 6), rats with BDL-induced early-stage liver fibrosis (n = 8), and rats with late-stage liver fibrosis (n = 11). The line indicates the median value in each group. Fold-changes were calculated using the 2^−ΔΔCt^ method. The Mann-Whitney test was used as the statistical significance test. (**E**) Liver fibrosis staging in rats subjected to BDL. HE: HE staining; masson: masson staining; VG: VG staining. The pictures were taken using a microscope (200x). (**A**) control group (F0); (**B**) stage 1 fibrosis (F1); (**C**) stage 2 fibrosis (F2); D, stage 3 fibrosis (F3); E, stage 4 fibrosis(F4). Representative data from three repeats are shown.

To examine the potential involvement of microRNAs in the initiation and progression of liver fibrosis, a miRNA expression profile of 12 blood RNA samples was generated using a microRNA microarray assay on the Paraflo™ platform (LC Sciences) and Sanger miRBase 18.0. (Fig. 1A). The results showed that miR-185 was significantly increased in early-stage fibrosis rats (F1-F2), and advanced-stage fibrosis rats (F3-F4), compared to the control. Most interestingly, the magnitude of the circulating miR-185 induction was dependent on the stages of fibrosis in DMN-induced liver fibrosis rat models (Fig. 1B,C). Similar results were consistently obtained in BDL-induced liver fibrosis rat models (Fig. 1D,E), although the magnitude of increases in the miRNA relative levels was less for DMN liver fibrosis than for BDL liver fibrosis. These results suggested the circulating miR-185 levels could be a potential marker for the pathogenesis and progression of liver fibrosis.

### Circulating miR-185 levels are elevated in patients with liver fibrosis in a stage-dependent manner

**Figure 2:**
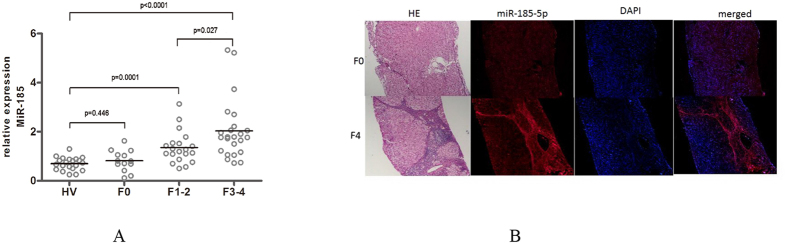
Elevated liver miR-185 expression blood and livers of patients with HBV-infected liver fibrosis. (**A**) Blood miR-185 levels in patients with HBV-infected liver fibrosis. The relative miR-185 levels were determined using qRT-PCR. Fold-changes were calculated using the 2^−ΔΔCt^ method. The Mann-Whitney test was performed for statistical significance. HV, healthy volunteers (n = 21); F0, patients that do not have liver fibrosis (n = 13); F1-F2, patients with early-stage fibrosis (n = 33); F3-F4, patients with advanced-stage fibrosis (n = 11). The line indicates the median value in each group. (**B**) Elevated liver miR-185 expression was located at sites of proliferation of myofibroblasts and deposition of collagen in patients with liver fibrosis. *In situ* hybridization (ISH) assay of miR-185-5p was performed using biopsy specimens from patients. Representative data from three repeats are shown.

To examine whether miR-185 levels are differentially altered in patients with liver fibrosis, we determined miR-185 levels in 21 healthy volunteer controls, 13 negative controls (F0), 20 HBV-infected early-stage fibrosis patients (F1-F2), and 24 HBV-infected advanced-stage fibrosis patients (F3-F4) using a TaqMan qRT-PCR(quantitative real-time PCR) assay. We found that miR-185 levels in patients with HBV-related fibrosis were significantly higher in early-stage fibrosis (fold-changes = 1.65, *p* = 0.0001) and advanced-stage fibrosis (fold-changes = 2.96, *P* < 0.0001) than those in the healthy control group (Fig. 2A). The levels of circulating miR-185 were higher in advanced-stage fibrosis compared with early-stage fibrosis (fold-changes = 1.79, P = 0.0267) (Fig. 2A). There was no obvious change in the blood levels of miR-185 in the F0 stage patients, compared to the healthy control group (*P* = 0.4460) (Fig. 2A). The results suggested that miR-185 blood levels were increased in HBV-infected patients with liver fibrosis. The magnitude of the increases was stage-dependent.

### The increases in the liver miR-185 levels were associated with collagen deposition

To examine the histological distribution of miR-185 in fibrotic livers, we performed an *in-situ* hybridization assay of miR185 in human liver fibrotic samples. The results showed that the miR-185 levels were higher in F4 staged liver fibrosis compared with F0 staged liver fibrosis (Fig. 2B).

### Circulating miR-185 levels are correlated with the progression of liver fibrosis but not HBV-DNA loads in patients

**Figure 3:**
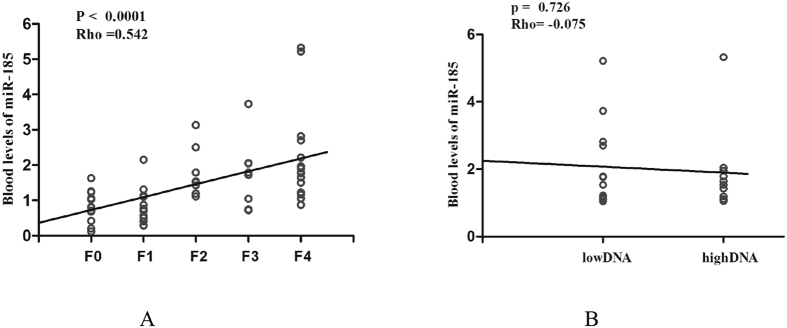
Circulating miR-185 levels were correlated with progression of liver fibrosis but not with viral loads in HBV-infected patients. (**A**) Circulating miR-185 levels were correlated with progression of liver fibrosis in HBV-infected patients. The fibrosis stages were confirmed using liver biopsy and histopathological characterization. The number of cases in each stage, F0 = 13, F1 = 12, F2 = 8, F3 = 8, or F4 = 16. The correlation was analyzed with the Spearman rank correlation coefficient test. (**B**) Circulating miR-185 levels were not correlated with viral loads in HBV-infected patients. Low DNA, patients with low viral loads (n = 11); high DNA, patients with high viral loads (n = 13). The correlations were analyzed with the Spearman rank correlation coefficient test.

To determine whether the miR-185 levels are correlated with the progression of liver fibrosis, we performed analysis of correlation between the miR-185 levels and the stages of liver fibrosis in 57 HBV-infected patients who underwent liver biopsy. These patients presented with different stages of histology-characterized fibrosis (F0 = 13, F1 = 12, F2 = 8, F3 = 8, and F4 = 16). Using linear regression and correlation analysis, we found that circulating miR-185 levels were positively correlated with fibrosis stages (Rho = 0.542, *p* < 0.001; Fig. 3A).

It has been shown that 10^4^ copies/mL or greater of circulating HBV-DNA is associated with a significant risk for progression to cirrhosis^18^,^19^. This suggested that the level of circulating serum HBV-DNA is a predictor of cirrhosis risk. To determine whether blood miR-185 levels are correlated with HBV viral loads, we analyzed correlation using samples from 24 HBV-infected patients with advanced-stage (F3-4) liver fibrosis. Thirteen patients had high viral loads (≥10^4^ copies/mL), and 11 had low viral loads (<10^4^ copies/mL). Using linear regression and correlation analysis, we found that there was no correlation between circulating miR-185 levels and HBV viral load (Rho = −0.075, *p* = 0.726; Fig. 3B).

### Diagnosis of liver fibrosis in patients with HBV infection using circulating miR-185 levels

**Figure 4:**
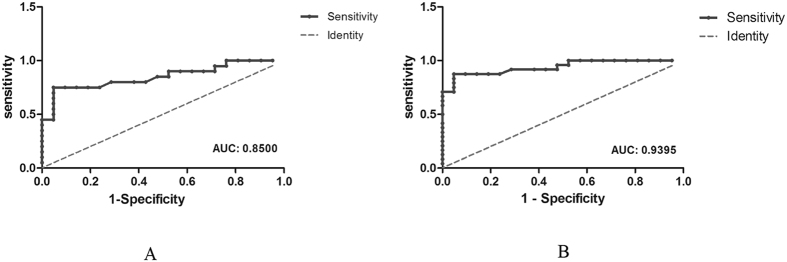
The blood miR-185 levels can discriminate (**A**) HBV-infected patients with early-stage fibrosis and (**B**) HBV-infected patients with advanced-stage fibrosis from healthy volunteers. The discrimination assay was performed using ROC analysis.

To determine the potential of circulating miR-185 levels in diagnosing liver fibrosis in patients with HBV infection, we performed SD (Sprague-Dawley, receiver-operator characteristic) analysis to evaluate the miR-185 levels for the detection of HBV-infected liver fibrosis progression using a cohort of 21 healthy volunteers, 20 patients with early-stage fibrosis, and 24 patients with advanced-stage fibrosis. The results showed that an area under the ROC curve (AUC) of 0.8500 (95% CI: 0.7274–0.9726, *P* = 0.0001) was observed for the miR-185 levels in the early-stage liver fibrosis. This could be used to specifically and sensitively discriminate patients with early-stage liver fibrosis from the healthy group (specificity 95.24%, sensitivity 75%, likelihood ratio 15.75, Fig. 4A). Moreover, miR-185 levels in advanced-stage fibrosis yielded an AUC of 0.9395 (95% CI: 0.8725-1.006, *P* < 0.0001), which could be used to discriminate advanced-stage fibrosis from healthy volunteers with a higher specificity (95.24%) and sensitivity (87.5%) (likelihood ratio 18.37) (Fig. 4B). The results suggested that the circulating miR-185 levels were useful in diagnosing liver fibrosis in patients with HBV infection with high specificity, sensitivity, and likelihood ratio.

### miR-185 over-expression resulted in increased expression of COL1A1 and a-SMA genes in HSC cells

**Figure 5:**
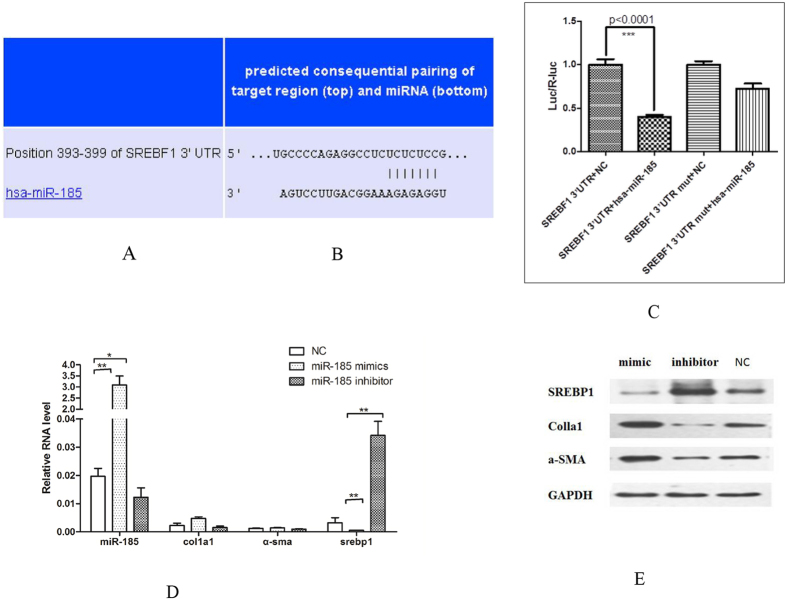
miR-185 targeted SREBF1 gene and changed COL1A1 and a-SMA mRNA and protein levels in HSC cells. (**A**) Predicted miR-185-targted site in SREBF1 gene. (**B**) Schematic constructs for miR-185 and miR-185 mutant plasmids. (**C**) SREBF1 gene expression was blocked by miR-185 but not miR-185mutant. 293T cells were transfected with reporter genes and miR-185 mimics and its inhibitors. A reporter gene assay was performed. (**D**,**E**) Effects of miR-185 on COL1A1 and a-SMA mRNA (**D**) and protein (**E**) levels in HSC cells. Messenger mRNA (**D**) and protein (**E**) levels were determined using qRT-PCR and Western blot assay, respectively. NC, negative control. Mimic, miRNA-185 mimics. Inhibitor, miRNA-185 inhibitor. Representative data from three repeats are shown.

We found that SREBF1 contained sites that were potentially targeted by miR-185 (Fig. 5A). To validate this finding, we performed a luciferase reporter gene assay with the human embryonic kidney cell line 293T, using constructs containing SREBF1 wild type or mutant sequences potentially targeted by miR-185 (Fig. 5B). The results showed that the luciferase activity of the wild-type construct was significantly decreased by miR-185, but not by the mutant constructs (Fig. 5C). The results confirmed that SREBF1 was the target of miR-185.

To determine the effect of induced expression of miR-185 on the expression of SREBF1, COL1A1 and a-SMA in HSC cells, we determined the levels of SREBF1, COL1A1 and a-SMA in HSC cells when over-expressing miR-185 mimic or its inhibitor. The results showed that miR-185 mimic decreased SREBF1 mRNA and protein levels in HSC cells (Fig. 5D,E). miR-185 mimic increased COL1A1 mRNA levels but had no obvious effect on a-SMA mRNA levels in HSC cells (Fig. 5D). miR-185 mimic significantly elevated COL1A1 and a-SMA protein levels in the HSC cells (Fig. 5E). These results suggested that miR-185 decreased SREBF1 expression and increased COL1A1 and a-SMA expression in HSC cells.

## Discussion

Liver fibrosis is reversible, depending on the pathological stage and an early intervention. Diagnosis at an early stage is a key step for early intervention of liver fibrosis. Traditional diagnosis using histopathological staining requires a biopsy, which is invasive and unsuitable for evaluating the effect of therapy after short intervals. An alternative approach is required to facilitate optimal post-treatment detection. In the current study, we evaluated the level of circulating miR-185 as a potential biomarker to predict initiation and progression of liver fibrosis using rat liver fibrosis models and specimens from patients. We found that miR-185 is significantly up-regulated in blood specimens from both HBV-related liver fibrosis patients and rats with liver fibrosis. Our *in situ* hybridization assay of miR-185 showed that miR-185 was located in collagen deposition regions during advanced liver fibrosis. The magnitude of increases in the miR-185 levels is correlated with the progressive stages of liver fibrosis but not with different viral loads in HBV-infected patients. The miR-185 levels can differentiate between early-staged and advanced-staged liver fibrosis in healthy controls with high specificity, sensitivity, and likelihood ratio. Furthermore, miR-185 targets SREBF1, and increases expression of COL1A1 and a-SMA genes that are involved in liver fibrosis. Our data confirm that circulating miR-185 is a potential biomarker for liver fibrosis. Since the samples we collected were blood samples from HBV liver disease patients, miR-185 is also as a biomarker for early diagnosis of HBV-related liver fibrosis.

In our current study, we have found that the expression of miR-185 is progressively elevated with aggravation of fibrosis. Interestingly, Wen and Han, *et al.*^20^ found that HCC is independently associated with an increase in miR-185^20^. miR-185 levels are increased in both liver fibrosis and HCC. Since liver fibrosis can progress to HCC, we speculate that there is an elevation in miR185 levels upon transition from liver fibrosis to HCC. Several serum-based methods and imaging methods have been developed to biomechanically measure hepatic fibrosis in some retrospective and prospective studies^2^. These non-invasive diagnostic modalities are excellent at identifying patients with cirrhosis; however, they do not accurately differentiate between the F2 and F3 stages of fibrosis^21^. Liver biopsy is still the gold standard for grading liver fibrosis; however, this invasive technique has a mortality rate of 0.1–0.01% and harbors the risk of severe complications^22^. Recent findings have indicated that circulating miRNAs could be potential biomarkers for liver disease^23^. For example, miR-181b in serum may be a potential diagnostic biomarker for cirrhosis^24^, and miR-19b was found to be up-regulated 4.3-fold in the serum of individuals with cirrhotic livers, compared with normal controls^18^. The expression of a list of serum 10 miRNAs such as *hsa-miR-4695-5p* and *hsa-miR-4651* were identified to be upregulated in livers with fibrosis, compared with those without fibrosis. It seemed that the levels of these miRNAs in the serum were unlikely correlated with the stages of fibrosis^25^. Nevertheless, there is currently no biomarker for the detection of the early–stage HBV-related liver fibrosis. In the current study, we investigated for the first time the feasibility of using blood miR-185 as an indicator for improved early diagnostic performance. Notably, the miR-185 levels can discriminate early-stage of HBV-related liver fibrosis from the healthy controls with high specificity (95.24%), sensitivity (75%), and likelihood ratio (15.75) using ROC analysis (Fig. 4A). The efficiency of diagnosis associated with the detection of miR-185 levels is greater than that of serum-based or imaging methods, which demonstrate lower specificity and sensitivity in the early diagnosis of liver fibrosis^2^,^21^. Therefore, circulating miR-185 levels may be a potential biomarker for the early-stage diagnosis of HBV-related liver fibrosis.

Studies have shown that cellular miR-185 levels are related to specific clinic-pathological features, such as vascular invasion, cell proliferation, and migration^26^,^27^,^28^. *In situ* hybridization data show that miR-185 is deposited in collagen deposition regions during advanced liver fibrosis, indicating that miR-185 levels may be induced in activated HSC cells. This is consistent with previous study. We speculate that increased miR-185 levels in the blood may be due to the increased release of miR-185 from the activated HSC cells.

It has been shown that SREBF1 inhibits HSC activation^29^,^30^. Inhibition of SREBF1 expression leads to increases in COL1A1 and a-SMA levels in cultured HSCs^31^. Induction of COL1A1 and a-SMA is a hallmark of HSC activation and liver fibrosis. In the current study, we found that miR-185 mimic inhibits SREBF1 expression and increases COL1A1 and a-SMA levels in HSC cells. Therefore, inhibition of SREBF1 expression following overexpression of miR-185 might be a mechanism associated with the increases in COL1A1 and a-SMA levels during liver fibrosis. This confirms that miR-185 is a promising indicator for liver fibrosis.

In conclusion, miR-185 levels can be used not only for the diagnosis of early-stage liver fibrosis but also as a potential biomarker for follow-up monitoring and assessment after treatment. To the best of our knowledge, this study is the first to investigate the feasibility of using peripheral blood miR-185 as an indicator of early-stage liver fibrosis. Our study, indicates that miR-185 levels might be used as a complemental diagnostic approach for the clinical pathological monitoring of early-stage liver fibrosis.

## Materials and Methods

### Patients and sample collection

A total of 78 blood samples were collected from 57 HBV-infected patients exhibiting different stages of fibrosis, admitted to Fuzhou Military General Hospital between September 2010 and May 2012. A cohort of 21 healthy volunteers were also recruited as part of this study. All of the subjects had previously been diagnosed as not having any other type of liver disease, and none had a history of alcohol abuse. The quantitative measurements of HBV DNA and other clinical and histological features of chronic HBV patients were assessed after liver biopsies. The liver fibrosis stage was determined according to the grade of the fibrosis (F0, F1, F2, F3, or F4, based on the METAVIR fibrosis staging system). Of the 57 patients, 13 were at stage F0, 20 were at F1-F2 (early stage), and 24 were at F3-F4 (advanced stage). Samples of fibrosis grade F0 were collected from chronic HBV-infected patients not presenting with liver fibrosis (negative controls). Table 1 shows the characteristics of HBV-infected patients at different clinical stages. The levels of HBV-DNA were determined using a Diagnostic Kit for Quantification of Hepatitis B Virus DNA (PCR-Fluorescene Probing) (Supbio Biotech, Guangzhou, China). The study was approved by the ethics committees of Changzheng hospital, China. The methods were carried out in accordance with the principles stated in the Declaration of Helsinki. Written informed consent was obtained from each patient.

### Animals

Male Sprague-Dawley (SD) rats (6–12 weeks old, body weight 200 ± 30 g,) were obtained from the Zoology Chinese Academy of Sciences (Shanghai, China) and fed in a SPF-graded facility. Standard rat chow and water were available ad libitum. The animal studies were approved by the ethics committees of the Second Military Medical University (Shanghai, China). The methods were carried out in accordance with the guidelines of the Institutional Animal Care and the Ethical Committee of the Second Military Medical University.

### Animal models and sample collection

For the DMN-induced liver fibrosis model, 42 male SD rats were randomly divided into five groups. The control group received a saline injection. Other groups received 1% DMN (i.p.) on the morning of every Monday, Tuesday, and Wednesday for four weeks.

For the BDL-induced liver fibrosis model, 34 male SD rats were disinfected and subjected to BDL. After the hepatic portal bile duct was opened, 1-cm of the bile duct was freed. The upper ends of the two lines and one of the lower ends was ligated. The ligated bile duct was cut in the middle to avoid bile duct recanalization. The animal received a penicillin injection two days after surgery and normal feeding for five weeks until completion of the experiment.

The DMN-induced hepatic fibrosis animal models were evaluated by a senior pathologist who assayed and judged the degree of liver fibrosis using hematoxylin and eosin (H&E) staining, Van Gieson(VG), and Masson’s trichrome staining.

After mice were anesthetized with 3% sodium pentobarbital (i.p., 0.1 ml/100 g body weight), liver tissues were collected. Blood samples (2.5 ml) were collected from the abdominal aorta using PAXgene Blood Collection Tube (BD, USA) and mixed by upside-down rotation at room temperature for at least two hours. After being stored at −20 °C for 24 hours, blood samples were then stored at −80 °C until required. Blood samples from 12 DMN-induced hepatic fibrosis rats were used for the miRNAs microarray assay on the Paraflo™ platform (LC Sciences) and Sanger miRBase 18.0.

### RNA preparation and qRT-PCR

Whole blood RNA was extracted following a previously published protocol^32^ using PAXgene Blood RNA Tube (BD, USA). The quality and quantity of RNA was assessed using a NanoDrop instrument. An A_260_/A_280_ ratio of approximately 2.0, and an A_260_/A_230_ ratio of greater than 1.5 were considered indicators of good quality RNA. RNA integrity was further verified by inspection of the 18S and 28S ribosomal RNA bands using denaturing gel electrophoresis. The levels of the blood miRNA were determined using TaqManq RT-PCR assays. All reagents, primers, and probes were purchased from Applied Bio-systems (Shanghai, China). The levels of the blood miRNA were determined using TaqManq RT-PCR assays.

RNA samples from cells were extracted using an RNeasy mini kit (Qiagen, Shanghai, China) according to the manufacturer’s protocol. The relative levels of genes colla1, α-sma, and SREBF1 were determined using a qRT-PCR assay with SYBR GREEN (Applied Bio-systems, Shanghai, China). U6 gene was used as an internal reference. The primers used to amplify these genes are listed in Table 2.

Quantitative real-time PCR was performed using an Applied Bio-Systems 7900HT thermo-cycler using the following conditions: 95 °C for 10 min, 40 cycles of 95 °C for 15 s and 60 °C for 60 s. The cycle threshold (CT) values were calculated using SDS software v2.4 (Applied Bio-systems, USA). The relative miRNA levels between the fibrosis models and the control were calculated using the 2^−ΔΔCt^ method.

### *In situ* hybridization (ISH) assay

ISH was performed using the miR-185-5p locked nucleic acid probe (5′-digoxigenin-UGGAGAGAAAGGCAGUUCCUGA-3′-digoxigenin) and the microRNA ISH Optimi-zation Kit (Exiqon, Vedbaek, Denmark) according to the manufacturer’s instructions. Briefly, deparaffinized arrays were incubated with 15 mg/mL proteinase K at 37 °C for 8 min. After dehydration, the slides were incubated with a 40 nmol/L miR-185-5p probe at 50 °C for 120 min, followed by stringent washes with 5X standard saline citrate, 1X standard saline citrate, and 0.2X standard saline citrate buffers at 50 °C, and finally incubated with digoxigenin blocking reagent (Roche, Mannheim, Germany). The signals were examined with a BX51 fluorescence microscope (Olympus, Japan).

### Western blot analysis

HSC cell samples were collected using RIPA cell lysis buffer after being washed three times with cold PBS. The protein concentration was determined using Coomassie Blue photometry. Proteins (15–30 μg/lane) were resolved on 10% SDS–polyacrylamide electrophoresis gels using a 10% stacking gel, followed by electro-transference to a nitrocellulose membrane (Millopore, Shanghai, China). The membranes were incubated with block buffer (Sangong, Shanghai, China), then incubated with a primary antibody (anti-COL1A1, cat# ab90395; anti-a-SMA, cat# ab5694; anti- SREBF1, cat# ab3259, abcam, Shanghai, China) in TBST buffer (Tris-HCl, pH 7. 4–7.6, 25 mM; NaCl 0.88%; KCl 0.02%; Tween 20 0.05%) for 2 h. After washing with TBST buffer, the peroxidase-conjugated secondary IgG was added and incubated in TBST buffer for 2 h. The membrane was covered and incubated with chemiluminescent reagents (Santa Cruz, CA, USA) after washing with TBST buffer, and then exposed to X-Ray films (Kodak, Shanghai, China). The films were developed using a Kodak Film Developer prior to visualization.

### Constructs of plasmids, transfection of cells, and reporter gene assay

The 3′ UTR segments of SREBF1, predicted to interact with miRNAs, were amplified by PCR from human genomic DNA and inserted into the pMIR-reporter vector (Applied Biosystems), using the *Mlu*I and *Hin*dIII sites immediately downstream from the luciferase stop codon.

For the transfection and luciferase reporter experiments, the human embryonic kidney cell line 293T was grown in 10% FBS in DMEM medium at 37 °C in a humidified atmosphere with 5% CO_2_. The cells were transfected in 96-well plates using Lipofectamine^®^ 2000 (Life Technologies) according to the manufacturer’s protocol. 0.2 μg of the firefly luciferase report vector and 0.01 μg of the control vector containing Renilla luciferase, pRL-CMV (Promega), or 100 nM miRNA oligonucleotides (Shanghai Gene Pharma Co. Ltd) were used for transfection in each well. Firefly and Renilla luciferase activities were determined consecutively using dual-luciferase assays (Promega) 48 h after transfection.

The miRNA mimics (ago-miR-185-5p), inhibitors (antago-miR-185-5p), and negative controls of miR-185-5p were purchased from RiboBio (RiboBio, Guangzhou, China). The cells were transfected with a 50 nM solution of the mimic, inhibitor, and negative controls. The Fu GENE HD transfection agent (Promega, USA) was used according to the manufacturer’s instructions.

### Statistical analysis

All values were expressed as the means ± standard deviation (SD) of at least three independent experiments. For the data obtained by qRT-PCR, the Mann-Whitney unpaired test was used for the comparison between the liver fibrosis samples and the controls. Receiver-operator characteristic (ROC) curves were established to evaluate the use of peripheral blood miRNA levels for discriminating patients with HBV fibrosis from healthy volunteers. The area under the ROC curve (AUC) was used as an accuracy index for evaluating the diagnostic performance of the selected miRNAs levels. Correlations were determined based on the Spearman rank correlation coefficient. All *p*-values were two sided, and *p* < 0.05 were considered statistically significant. All statistical calculations were performed with SPSS 17.0 (IBM, Chicago, IL, USA). Graphs were generated using GraphPad Prism 5.0 (GraphPad Software, Inc., CA, USA).

## Additional Information

**How to cite this article**: Li, B.-b *et al.* Potentials of the elevated circulating miR-185 level as a biomarker for early diagnosis of HBV-related liver fibrosis. *Sci. Rep.* **6**, 34157; doi: 10.1038/srep34157 (2016).

## Figures and Tables

**Table 1:** Patients with F0-, F1/F2-, F3/F4- staged liver fibrosis.

Clinical data	Health	F0	F1/F2	F3/F4
Sex (M/F)	13/8	11/2	14/6	17/7
Age (years)	42.3 ± 4.3	28 ± 7.6	40.9 ± 3.5	46.3 ± 6.3
ALT (μg/l)	12.6 ± 7.8	13.0 ± 8.1	42.3 ± 16.3	43.0 ± 11.3
AST (μg/l)	15.6 ± 2.9	15.9 ± 2.6	63.7 ± 13.9	60.5 ± 18.3
Albumin (g/dl)	74.2 ± 3.1	66 ± 2.8	47.2 ± 10.1	32.4 ± 16.5
HBV-DNA (log10copies/ml)	0	5.3 ± 1.1	4.8 ± 2.3	5.6 ± 1.7
Anti-virus therapy	No	No	No	No
Stage (NC/F0/F1-F2/F3-4)	21/0/0/0/	0/13/0/0/	0/0/20/0	0/0/0/24

**Table 2:** Gene-specific primers for the qRT-PCR assay.

Gene	Primer Sequences (5′-3′)	Product	T_m_(^o^C)
col1a1	sense: GCTCGTGGATTGCCTGGAACAG	235 bp	55
	antisense: CACCGACAGCACCATCGTTACC		
α-sma	sense: GCCACTGCTGCTTCCTCTTCTT	136 bp	55
	antisense: CCGCCGACTCCATTCCAATGAA		
SREBF1	sense: AGGAGAACCTGACCCTGCGAAG	214 bp	55
	antisense: TCACTGCCACCACTGCTGCT		
U6	sense: CTCGCTTCGGCAGCACATATACT		
	Antisense: ACGCTTCACGAATTTGCGTGTC		
